# Is conventional functional liver remnant volume higher than 40% still sufficient to prevent post‐hepatectomy liver failure in jaundiced patients with hilar cholangiocarcinoma? A single‐center experience in China

**DOI:** 10.1002/cam4.7342

**Published:** 2024-07-05

**Authors:** Tian‐Run Lv, Wen‐Jie Ma, Fei Liu, Hai‐Jie Hu, Yan‐Wen Jin, Fu‐Yu Li

**Affiliations:** ^1^ Department of Biliary Tract Surgery, General Surgery West China Hospital of Sichuan University Chengdu Sichuan China; ^2^ Research Center for Biliary Diseases West China Hospital of Sichuan university Chengdu Sichuan China

**Keywords:** functional liver remnant volume, hilar cholangiocarcinoma, liver failure, obstructive jaundice

## Abstract

**Objective:**

Our study aims to evaluate the predictive accuracy of functional liver remnant volume (FLRV) in post‐hepatectomy liver failure (PHLF) among surgically‐treated jaundiced patients with hilar cholangiocarcinoma (HCCA).

**Methods:**

We retrospectively reviewed surgically‐treated jaundiced patients with HCCA between June, 2000 and June, 2018. The correlation between FRLV and PHLF were analyzed. The optimal cut off value of FLRV in jaundiced HCCA patients was also identified and its impact was furtherly evaluated.

**Results:**

A total of 224 jaundiced HCCA patients who received a standard curative resection (43 patients developed PHLF) were identified. Patients with PHLF shared more aggressive clinic‐pathological features and were generally in a more advanced stage than those without PHLF. An obvious inconsistent distribution of FLRV in patients with PHLF and those without PHLF were detected. FLRV (continuous data) had a high predictive accuracy in PHLF. The newly‐acquired cut off value (FLRV = 53.5%, sensitivity = 81.22%, specificity = 81.4%) showed a significantly higher predictive accuracy than conventional FLRV cut off value (AUC: 0.81 vs. 0.60, *p* < 0.05). Moreover, patients with FLRV lower than 53.5% also shared a significantly higher major morbidity rate as well as a worse prognosis, which were not detected for FLRV of 40%.

**Conclusion:**

For jaundiced patients with HCCA, a modified FLRV of 53.5% is recommended due to its great impact on PHLF, as well as its correlation with postoperative major morbidities as well as overall prognosis, which might help clinicians to stratify patients with different therapeutic regimes and outcomes. Future multi‐center studies for training and validation are required for further validation.

## 1 INTRODUCTION

As for the management of hilar cholangiocarcinoma (HCCA), the curative‐intent surgery, including an extra‐hepatic bile duct resection combined with partial hepatectomy and a regional or more extended lymphadenectomy, has always been regarded as the gold standard therapeutic modality.^1^, ^2^, ^3^ However, such invasive surgical procedure often takes a high risk of early mortalities, especially in those who developed post‐hepatectomy liver failure (PHLF). One recently‐published meta‐analysis revealed a 12% 90‐day mortality rate and a 40% major morbidity rate in patients with HCCA who have received a radical resection.^4^ A higher mortality rate reaching 18% was even observed in some experienced centers.^4^

Accumulating evidence has suggested that the preoperatively‐estimated functional liver remnant volume (FLRV) had the most intensive relationship with PHLF among resected HCCA patients who received a combined partial hepatectomy at the same time.^5^, ^6^, ^7^, ^8^, ^9^, ^10^, ^11^ A better‐preserved functional liver remnant after partial hepatectomy can be extremely vital due to its key role in patients' postoperative rehabilitation, accelerating anastomotic growth and the further prevention of early occurrence of anastomotic leakage. Currently, the classical and acceptable criteria of FLRV is larger than 40%, which has been validated in previous studies.^8^, ^12^ However, this standard has its limitations that FLRV is only defined broadly but not specifically. For example, for patients with cirrhosis or obstructive jaundice who shared a relatively worse liver health status, whether FLRV should be modified accordingly remains unexplored. Patients with HCCA were often presented with obstructive jaundice preoperatively and suffered chronic injuries resulted from the elevated serum bilirubin level‐mediated hepatocytotoxicity. Compared with those without obstructive jaundice who were about to receive curative surgery, jaundiced patients generally shared an impaired liver function reserve. Therefore, in such a circumstance, a modified FLRV in jaundiced patients with HCCA who were about to receive curative surgery might be more appropriate and feasible. However, having screened all relevant studies, corresponding studies focused on the optimal FLRV in jaundiced HCCA patients who were about to receive a standard surgical procedure were still lacking.

Therefore, current study was performed to evaluate the predictive value of FLRV for postoperative liver failure among resected HCCA patients who have received a combined partial hepatectomy at the same time. Moreover, an optimal cut off value would also been furtherly identified. The predictive accuracy of newly‐acquired predictive criteria of FLRV versus the classical FLRV (40%) would also be compared.

## 2 METHODS

### 2.1 Patients

We retrospective reviewed resected HCCA patients between June, 2000 and June, 2018 at our hospital. Only patients received a standard radical resection were included, including an extra‐hepatic bile duct resection, combined with partial hepatectomy and lymphadenectomy. A right or left hemi‐hepatectomy or more extended hepatectomy would be performed in patients with Bismuth III or IV disease with an obvious liver invasion. For patients with Bismuth I or II disease, when an obvious caudate lobe invasion was detected, combined partial hepatectomy would also be performed. Moreover, a wedge resection would also be performed in order to achieve cholangioplasty and the subsequent high position biliary‐enteric anastomosis. This study was approved by institutional ethics review board of West China Hospital.

### 2.2 Preoperative evaluation, preparation, and surgery

The perioperative management were consistent with our previous studies.^13^, ^14^, ^15^, ^16^ Upon admission, a systemic body examination, including peripheral blood‐based examination such as tumor‐specific biomarkers CA199, or radiological examinations such as computed tomography (CT) or magnetic resonance imaging (MRI), would be applied in all patients to have an initial evaluation on tumor stage and tumor load. Patients who were considered to have developed distant metastatic disease would be excluded. A preoperative percutaneous trans‐hepatic cholangial drainage or endoscopic biliary drainage, combined with hepatoprotective therapy, would be applied in those with obstructive jaundice (TB level >200 μmol/L) so as to relieve the high bilirubin‐mediated hepatotoxicity as well as prevent the occurrence of PHLF. After drainage, patients with preoperative bilirubin level lower than 200 μmol/L were considered eligible for curative surgery. The liver volumetry has been regularly performed in our center since the first hepatectomy in the general surgery department. If the estimated FLRV indicated a relative compensable liver volume after partial hepatectomy, PVE would be avoided and more aggressive surgical procedures would be acquired, especially a wider resection margin would be performed in order to ensure tumor clearance. However, for patients whose FLRV were less than 40% or remnant liver volume seemed to be un‐compensable after surgery, the potential risk of PHLF, ICU admission with an extremely increased hospital expense, or even perioperative mortalities would be informed in detail to patients and their families. If they still insisted on performing curative surgery, they were required to sign an informed consent form that they were willing to undertake the relevant risk. The FLRV was mainly assessed and calculated via computed tomography‐volumetric evaluation. Moreover, a preoperative Child A or B degree of liver function was also required. The specific exact inclusion and exclusion criteria were consistent with our previous series.^16^ All surgical candidates have experienced a multi‐disciplinary discussion to find the most applicable surgical regime.

### 2.3 The definition of PHLF

Regarding the criteria of PHLF, current study adopted the criteria proposed by the International Hepatic Surgery Research Group (ISGLS) in 2011.^17^ As is illustrated in Figure S2, for cases with preoperative normal INR level (≤1.5), a higher or abnormal INR level (>1.5) combined with hyper‐bilirubinemia level (>17.1 μmol/L) were diagnosed with PHLF.^17^ However, for cases with obstructive jaundice derived from various postoperative factors, such as cholangitis, the criteria mentioned above would be not feasible. For cases with preoperatively‐detected abnormal INR level (>1.5), an increased INR level than preoperative INR level combined with hyper‐bilirubinemia level (>17.1 μmol/L) were diagnosed with PHLF. Additionally, for cases with a normal INR on or after post‐surgical Day 5, patients with a hyperbilirubinemia combined with coagulation disfunction, such as the requirement of continuous infusion of fresh frozen plasma for the stability of coagulation function, would also be considered. The severity of PHLF is classified as three categories: Grade A: only with laboratory parameters abnormalities and requires no special clinical care; Grade B: with laboratory parameters abnormalities but requires special clinical care or non‐invasive intervention; Grade C: with laboratory parameters abnormalities but requires invasive intervention, such as artificial liver, or liver transplantation.^17^

### 2.4 Follow up

All surgically‐treated patients were informed to monitor their disease status at the outpatient of our hospital every 1–2 months in the first year and every six months thereafter. Our researchers would renew their survival data once a year via telephone or any other social media. The most‐recently follow‐up was performed on October 1 2023.

### 2.5 Statistical analysis

IBM SPSS version 25.0 (IBM SPSS, Chicago, IL, USA), Graph‐Pad Prism 7, and R software (version 4.2.2) (*R package pROC*) were used for statistical analysis. All data were recorded as categorical data, which were presented as numbers (percentages) and compared via chi squared and Fisher's exact tests. Overall survival (OS) was defined as the survival period from the date of receiving radical surgery to the date of death or last follow‐up. Disease‐free survival (DFS) was defined as the survival period from the date of receiving surgery to the date of recurrence or disease progression. Kaplan–Meier curves were used for evaluating survival differences. Cox‐proportional hazards model was used to create multivariate model for independent prognostic factors for survival, which were presented with hazard ratio (HR) with its 95% confidence interval (CI). The binary logistic regression was used for the selection of predictors as well as independent predictors on PHLF, which were presented with odds ratio (OR) and its 95% CI. *p* values lower than 0.05 indicated the existence of statistical significance.

## 3 RESULTS

### 3.1 Comparative analyses between patients with PHLF and those without PHLF in terms of clinic‐pathological features and long‐term survival

Based our own cancer database, a total of 380 HCCA patients received curative surgery in our hospital and 90 patients did not receive a combined hepatectomy. Among the remaining 290 patients who received a combined hepatectomy, 54 patients were presented without jaundice and were therefore excluded. Moreover, owing to the inadequate original data, 12 patients were furtherly excluded. Finally, a total of 224 jaundiced patients who received the standard curative surgery with adequate clinical data were finally identified. As is recorded in Table 1, patients who developed PHLF and those who did not develop PHLF were comparable in age (≥70 vs. <70) (*p* = 0.1630) and sex (male vs. female) (*p* = 0.2650). However, patients in the former group were more frequently detected with Bismuth III‐IV disease (79.1% vs. 54.1%, *p* = 0.0420). Moreover, patients with PHLF shared a higher but not statistically significant incidence of receiving preoperative biliary drainage (95.3% vs. 88.4%, *p* = 0.1630). Pre‐surgery PVE was more frequently performed in patients with PHLF (18.6% vs. 3.9%, *p* = 0.0010). Major hepatectomy (48.8% vs. 17.1%, *p* < 0.0001) and major vascular resection and reconstruction (41.9% vs. 26%, *p* = 0.0320) were more frequently performed in patients with PHLF. Patients who developed PHLF were generally in a more advanced TNM stage (III–IV disease: 90.7% vs. 74.6%, *p* = 0.0140). The proportion of patients with FLRV <40% was significantly higher in patients with PHLF (32.6% vs. 13.3%, *p* = 0.0040). The incidence of concurrent other postoperative major morbidities (Clavien‐Dindo grade III–IV) was significantly higher in patients with PHLF (52.1% vs. 12.2%, *p* < 0.0001). Comparable incidence of mortalities within 90 days after surgery (*p* = 0.1570) and overall recurrence rate (*p* = 0.2180) were acquired between two group.

**TABLE 1:** Baseline characteristics of the entire cohort based on liver failure.

Variables	Patients with liver failure (*n* = 43)	Patients without liver failure (*n* = 181)	*p* value
Age			0.1630
≥70	35 (81.4%)	160 (88.4%)	
<70	8 (18.6%)	21 (11.6%)	
Sex			0.2650
Male	29 (67.4%)	110 (60.8%)	
Female	14 (32.6%)	71 (39.2%)	
Bismuth type			0.0420
I–II	9 (20.9%)	65 (35.9%)	
III–IV	34 (79.1%)	116 (54.1%)	
Concurrent hepatatrophy			0.2650
Yes	14 (32.6%)	71 (39.2%)	
No	29 (67.4%)	110 (60.8%)	
Concurrent hepatitis			0.3670
Yes	4 (9.3%)	8 (4.4%)	
No	39 (90.7%)	173 (95.6%)	
Concurrent cholangitis			0.3560
Yes	5 (11.6%)	28 (15.5%)	
No	38 (88.4%)	153 (84.5%)	
Preoperative biliary drainage			0.1630
Performed	41 (95.3%)	160 (88.4%)	
Not performed	2 (4.7%)	21 (11.6%)	
Preoperative PVE			0.0010
Yes	8 (18.6%)	7 (3.9%)	
No	35 (81.4%)	174 (96.1%)	
Preoperative CA199			0.5350
>150 U/mL	26 (60.5%)	100 (55.2%)	
≤150 U/mL	17 (39.5%)	81 (44.8%)	
Preoperative liver function (child class)			0.3690
Child A	4 (9.3%)	12 (6.6%)	
Child B	39 (90.7%)	169 (93.4%)	
Lymphadenectomy			0.9680
Regional	34 (79.1%)	140 (77.3%)	
Extended	9 (20.9%)	41 (22.7%)	
Major vascular reconstruction^a^			0.0320
Performed	18 (41.9%)	47 (26%)	
Not performed	25 (58.1%)	134 (74%)	
Major hepatectomy			<0.0001
Performed	21 (48.8%)	31 (17.1%)	
Not performed	22 (51.2%)	150 (82.9%)	
Surgical margin			0.2410
Negative	31 (72.1%)	142 (78.5%)	
Positive	12 (27.9%)	39 (21.5%)	
Surgical margin width			0.1500
≤5 mm	14 (32.6%)	40 (22.1%)	
>5 mm	29 (67.4%)	141 (77.9%)	
Resected tumor size			0.6150
≤2 cm	13 (30.2%)	62 (34.3%)	
>2 cm	30 (69.8%)	119 (65.7%)	
Perineural invasion			0.5510
Yes	24 (55.8%)	110 (60.8%)	
No	19 (44.2%)	71 (39.2%)	
Lymph‐vascular invasion			0.1750
Yes	3 (7.0%)	30 (16.6%)	
No	40 (93.0%)	151 (83.4%)	
Lymph node status (8th AJCC)			0.3140
N−	24 (55.8%)	116 (64.1%)	
N+	19 (44.2%)	65 (35.9%)	
T stage (8th AJCC)			0.1290
T1–2	10 (23.3%)	64 (35.4%)	
T3–4	33 (76.7%)	117 (64.6%)	
TNM stage (8th AJCC)			0.0140
I/II	4 (9.3%)	46 (25.4%)	
III/IV	39 (90.7%)	135 (74.6%)	
FLRV (%)			0.0040
<40%	14 (32.6%)	24 (13.3%)	
≥40%	29 (67.4%)	157 (86.7%)	
Postoperative major morbidity (C–D Grade III–IV)			<0.0001
Yes	28 (65.1%)	22 (12.2%)	
No	15 (34.9%)	159 (77.8%)	
Mortalities within 90 days			0.1570
Yes	3 (7.0%)	3 (1.7%)	
No	40 (93.0%)	178 (98.3%)	
Recurrence			0.2180
Yes	21 (48.8%)	74 (40.9%)	
No	22 (51.2%)	107 (59.1%)	

Abbreviations: AJCC, American Joint on Cancer; AJCC, American Joint Committee on Cancer; C–D grade, Clavien‐Dindo grade; FLRV, functional liver remnant volume; PVE, portal vein embolization.

^a^ Resected more than three segments.

**FIGURE 1:**
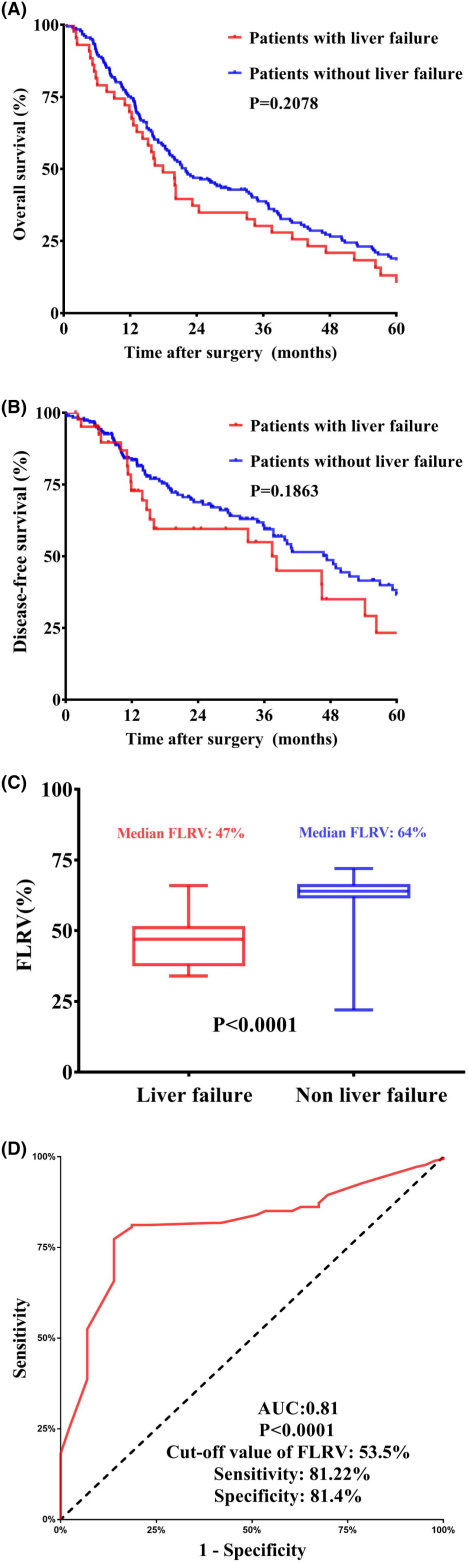
KM curves between patients who developed PHLD and those without PHLD. (A) OS; (B) DFS. The inconsistent distribution of FLRV between patients with PHLD and those without PHLD as well as the predictive accuracy of FLRV (continuous data) was also presented. (C) Box plot showing the inconsistent distribution of FLRV; (D) ROC curve reflecting the predictive accuracy of FLRV.

In the survival analyses, as is illustrated in Figure 1, patients with PHLF shared comparable OS (median survival period: 17.9 vs. 22.02 months, *p* = 0.2078) and DFS (median survival period: 37.44 vs. 47.33 months, *p* = 0.1863) versus those without PHLF.

### 3.2 Predictive accuracy of FLRV (continuous) in PHLF and the cut off value and the subgroup analyses based on major hepatectomy

As is presented in Figure 1C, the level of FLRV in patients with PHLF and those without PHLF varied a lot (*p* < 0.0500). We subsequently performed a ROC curve evaluating the predictive accuracy of FLRV in predicting the occurrence of PHLF among jaundiced post‐hepatectomy HCCA patients. The result indicated a high predictive accuracy (AUC: 0.81, *p* < 0.0001) (Figure 1D). Moreover, we also acquired the optimal cut off value of FLRV in jaundiced HCCA patients with favorable specificity and sensitivity. The results indicated that the optimal cut off value of FLRV was 53.5%, with a sensitivity of 81.2% and a specificity of 81.4% (Figure 1D). At the same time, we evaluated the predictive value of FLRV (continuous data) in PHLF. The results suggested that FLRV (continuous data) was an independent predictor of PHLF (*p* < 0.0001) (Table S1). We furtherly evaluated the predictive accuracy of FLRV in PHLF among jaundiced HCCA patients based on the performance of major hepatectomy. For patients who received major hepatectomy, FLRV failed to show a significant predictive accuracy (AUC: 0.52) (Figure S1A). However, for patients who received partial hepatectomy but not major hepatectomy, FLRV revealed a significant predictive accuracy (AUC: 0.86) (Figure S1B).

### 3.3 Comparative analyses regarding clinic‐pathological features and long‐term survival based on different FLRV cut off values

As is recorded in Table 2, we furtherly evaluated the impact of newly‐acquired FLRV cut off value in terms of clinic‐pathological features and long‐term survival. Patients with FLRV <53.5% were comparable versus patients with FLRV ≥53.5% in terms of age (*p* = 0.3730) and sex (*p* = 0.9560). The proportion of patients with Bismuth III‐IV disease was significantly higher in patients with FLRV <53.5% (*p* = 0.0070). Concurrent hepato‐atrophy was more frequently detected in patients with FLRV<53.5% (*p* = 0.0420). Preoperative PVE was more frequently performed in patients with FLRV <53.5% (*p* < 0.0001). Major hepatectomy (*p* < 0.0001) and major vascular resection and reconstruction (*p* = 0.0010) were also more frequently performed among patients in the former group. Comparable surgical margin status (*p* = 0.2560), resected margin width (*p* = 0.2550) and resected tumor size (*p* = 0.3410) were acquired. Comparable pathological features, such as perineural invasion, were also acquired between two groups. Patients with FRLV <53.5% were generally in a more advanced stage. Moreover, higher frequencies of postoperative any morbidity (*p* = 0.0010), major morbidities (*p* < 0.0001), and PHLF (*p* < 0.0001) were detected among patients with FLRV <53.5%. Comparable incidence of mortalities within 90 days after surgery (*p* = 0.1390) and overall recurrence rate (*p* = 0.6110) were acquired between two group. In the survival analyses, FLRV of 53.5% could furtherly stratify patients with different survival outcomes that jaundiced HCCA patients with FLRV <53.5% shared a much worse OS (median survival period: 14.97 vs. 26.19 months, *p* = 0.0038) (Figure 3A) but similar DFS (median survival period: 37.44 vs. 47.33 months, *p* = 0.3194) (Figure 3B) than those with FLRV ≥53.5%.

**TABLE 2:** Basic characteristics of patients according to cut off values of FLRV.

Variables	No. patients		No. patients		*p* value
	FLRV <53.5% (*n* = 69)	FLRV ≥53.5% (*n* = 155)	*p* value	FLRV <40% (*n* = 38)		FLRV ≥40% (*n* = 186)
Age			0.3730			0.5670
<70	58 (84.1%)	137 (88.4%)		32 (84.2%)	163 (87.6%)	
≥70	11 (15.9%)	18 (11.6%)		6 (15.8%)	23 (12.4%)	
Sex			0.9560			0.8310
Male	43 (62.3%)	96 (61.9%)		23 (60.5%)	116 (62.4%)	
Female	26 (37.7%)	59 (38.1%)		15 (39.5%)	70 (37.6%)	
Bismuth type			0.0070			0.0850
I–II	14 (20.3%)	60 (38.7%)		8 (21.1%)	66 (35.5%)	
III–IV	55 (79.7%)	95 (61.3%)		30 (78.9%)	120 (64.5%)	
Concurrent hepatatrophy			0.0420			0.1890
Yes	33 (47.8%)	52 (33.5%)		18 (47.4%)	67 (36.0%)	
No	36 (52.2%)	103 (66.5%)		20 (52.6%)	119 (64.0%)	
Concurrent hepatitis			0.4650			0.6720
Yes	3 (4.3%)	9 (5.8%)		1 (2.6%)	11 (5.9%)	
No	66 (95.7%)	146 (94.2%)		37 (97.4%)	175 (94.1%)	
Concurrent cholangitis			0.1960			0.5810
Yes	7 (10.1%)	26 (16.8%)		4 (10.5%)	29 (15.6%)	
No	62 (89.9%)	129 (83.2%)		34 (89.5%)	157 (84.4%)	
Preoperative biliary drainage			0.3880			0.4610
Yes	64 (92.8%)	138 (89.0%)		36 (94.7%)	166 (89.2%)	
No	5 (7.2%)	17 (11.0%)		2 (5.3%)	20 (10.8%)	
Preoperative PVE			<0.0001			0.0560
Yes	14 (20.3%)	1 (0.6%)		0 (0.0%)	15 (8.1%)	
No	55 (79.7%)	154 (99.4%)		38 (100.0%)	171 (91.9%)	
Preoperative CA199			0.0170			0.0170
>150 U/mL	47 (68.1%)	79 (51.0%)		28 (73.7%)	98 (52.7%)	
≤150 U/mL	22 (31.9%)	76 (49.0%)		10 (26.3%)	88 (47.3%)	
Preoperative liver function (child class)			0.8100			0.4010
Child A	4 (5.8%)	12 (7.7%)		1 (2.6%)	15 (8.1%)	
Child B	65 (94.2%)	143 (92.3%)		37 (97.4%)	171 (91.9%)	
Lymphadenectomy			0.5790			0.5160
Regional	52 (75.4%)	122 (78.7%)		28 (73.7%)	146 (78.5%)	
Extended	17 (24.6%)	33 (21.3%)		10 (26.3%)	40 (21.5%)	
Major vascular reconstruction^a^			0.0010			0.2440
Performed	30 (43.5%)	35 (22.6%)		14 (36.8%)	51 (27.4%)	
Not performed	39 (56.5%)	120 (77.4%)		24 (63.2%)	135 (72.6%)	
Major hepatectomy			<0.0001			<0.0001
Performed	52 (75.4%)	0 (0.0%)		35 (92.1%)	17 (9.1%)	
Not performed	17 (24.6%)	155 (100.0%)		3 (7.9%)	169 (90.9%)	
Surgical margin			0.2560			0.7820
Negative	50 (72.5%)	123 (79.4%)		30 (78.9%)	143 (76.9%)	
Positive	19 (27.5%)	32 (20.6%)		8 (21.1%)	43 (23.1%)	
Surgical margin width			0.2550			0.4440
≤5 mm	20 (29.0%)	34 (21.9%)		11 (28.9%)	43 (23.1%)	
>5 mm	49 (71.0%)	121 (88.1%)		27 (71.1%)	143 (76.9%)	
Resected tumor size			0.3410			0.1600
≤2 cm	20 (29.0%)	55 (35.5%)		9 (23.7%)	66 (35.5%)	
>2 cm	49 (71.0%)	100 (64.5%)		29 (76.3%)	120 (64.5%)	
Perineural invasion			0.2070			0.1750
Yes	37 (53.6%)	97 (62.6%)		19 (50.0%)	115 (61.8%)	
No	32 (46.4%)	58 (37.4%)		19 (50.0%)	71 (38.2%)	
Lymph‐vascular invasion			0.4540			0.0880
Yes	12 (17.4%)	21 (13.5%)		9 (23.7%)	24 (12.9%)	
No	57 (82.6%)	134 (86.5%)		29 (76.3%)	162 (77.1%)	
Lymph node status (8th AJCC)			0.7940			0.2320
N−	44 (63.8%)	96 (61.9%)		11 (28.9%)	73 (39.2%)	
N+	25 (36.2%)	59 (38.1%)		27 (71.1%)	113 (60.8%)	
T stage (8th AJCC)			0.0070			0.3340
T1–2	14 (20.3%)	60 (38.7%)		10 (26.3%)	64 (34.4%)	
T3–4	55 (79.7%)	95 (61.3%)		28 (73.7%)	122 (65.6%)	
TNM stage (8th AJCC)			0.0600			0.8370
I–II	10 (14.5%)	50 (32.3%)		8 (21.1%)	42 (22.6%)	
III–IV	59 (85.5%)	115 (67.7%)		30 (78.9%)	144 (77.4%)	
Postoperative any morbidity			0.0010			0.2320
Yes	54 (78.3%)	86 (55.5%)		27 (71.1%)	113 (60.8%)	
No	15 (21.7%)	69 (44.5%)		11 (28.9%)	73 (39.2%)	
Postoperative major morbidity (C–D Grade III–IV)			<0.0001			0.1330
Yes	27 (39.1%)	23 (14.8%)		12 (31.6%)	38 (20.4%)	
No	42 (60.9%)	132 (85.2%)		26 (68.4%)	148 (79.6%)	
Liver failure			<0.0001			0.0020
Yes	35 (50.7%)	8 (5.2%)		14 (36.8%)	29 (15.6%)	
No	34 (49.3%)	147 (94.8%)		24 (63.2%)	157 (84.4%)	
Mortalities within 90 days			0.1390			1.0000
Yes	4 (5.8%)	2 (1.3%)		1 (2.6%)	5 (2.7%)	
No	65 (94.2%)	153 (98.7%)		37 (97.4%)	181 (97.3%)	
Recurrence			0.6110			0.4970
Yes	31 (44.9%)	64 (41.8%)		18 (47.4%)	77 (41.4%)	
No	38 (55.1%)	91 (58.2%)		20 (52.6%)	109 (58.6%)	

Abbreviations: AJCC, American Joint Committee on Cancer; C–D grade, Clavien‐Dindo grade; FLRV, functional liver remnant liver volume; PVE, portal vein embolization.

^a^ Resected more than three segments.

As for the other conventional FLRV cut off value of 40%, similar observations were also detected. Patients with FLRV <40% and those with FLRV ≥40% were comparable in terms of Bismuth type (*p* = 0.0850), concurrent hepato‐atrophy (*p* = 0.1890), and preoperative PVE (*p* = 0.0560). Comparable incidence of major vascular resection and reconstruction (*p* = 0.2440) were also acquired between two groups. The overall tumor stage was also comparable between two groups (*p* = 0.8370). Moreover, comparable incidence of postoperative any complication (*p* = 0.2320) and major complication (*p* = 0.1330) were detected between two groups. FLRV of 40% had no impact on the overall prognosis that comparable OS (median survival period: 17.07 vs. 22 months, *p* = 0.3194) (Figure 3C) and DFS (median survival period: 37.44 vs. 46.73 months, *p* = 0.3315) (Figure 3D) were acquired between two groups.

### 3.4 Comparison of the predictive accuracy of PHLF between FLRV of 53.5% and FLRV of 40% among resected HCCA patients with jaundice

**FIGURE 2:**
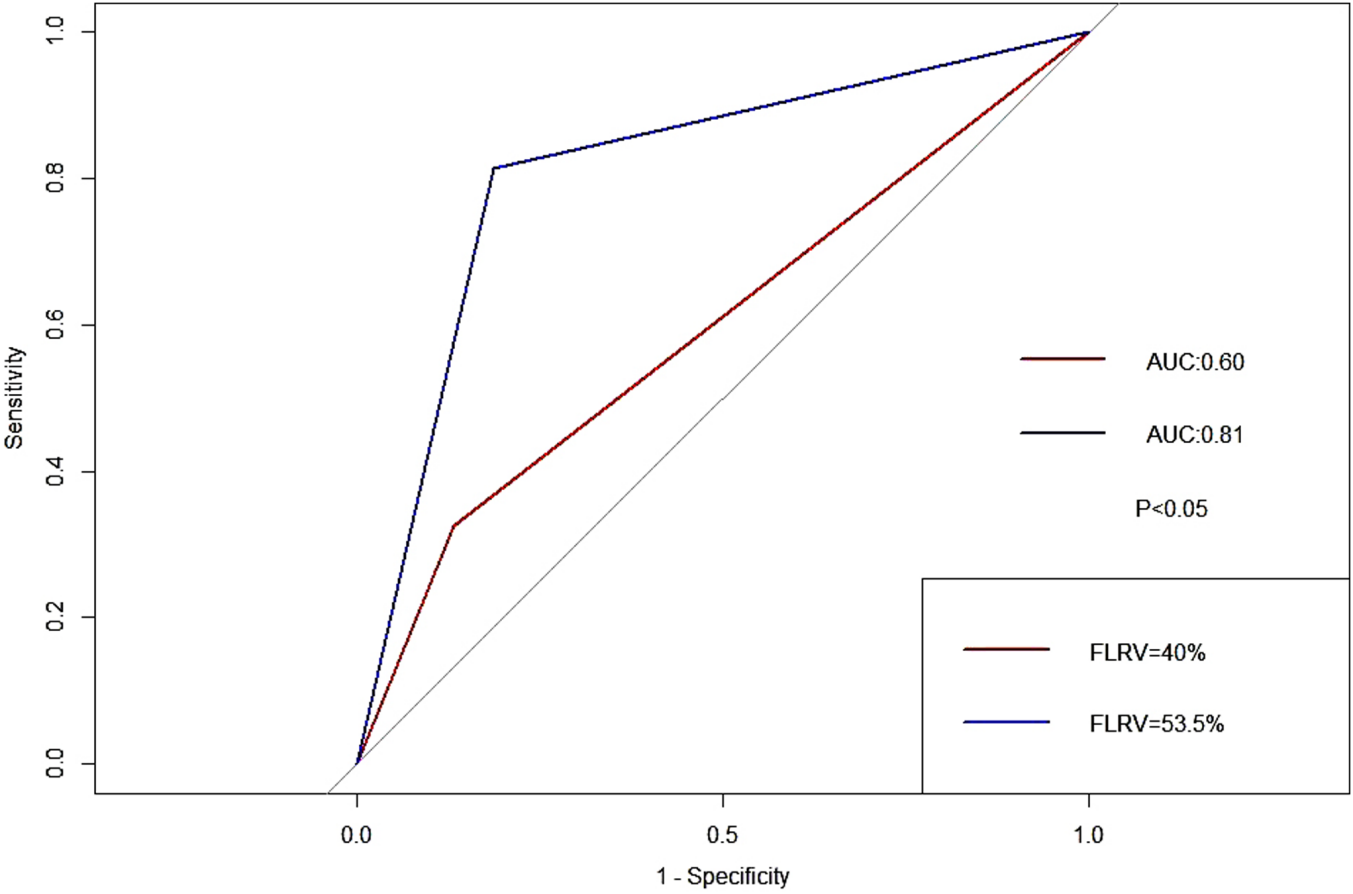
ROC curve comparing the predictive accuracy of PHLF among jaundiced HCCA patients based on different cut off value of FLRV.

We furtherly compared the predictive accuracy of newly‐acquired FLRV criteria (53.5%) versus conventional FLRV criteria (40%) in PHLF. The result indicated a significantly higher predictive accuracy of our new criteria (AUC: 0.81 vs. 0.60, *p* < 0.0500) (Figure 2).

**FIGURE 3:**
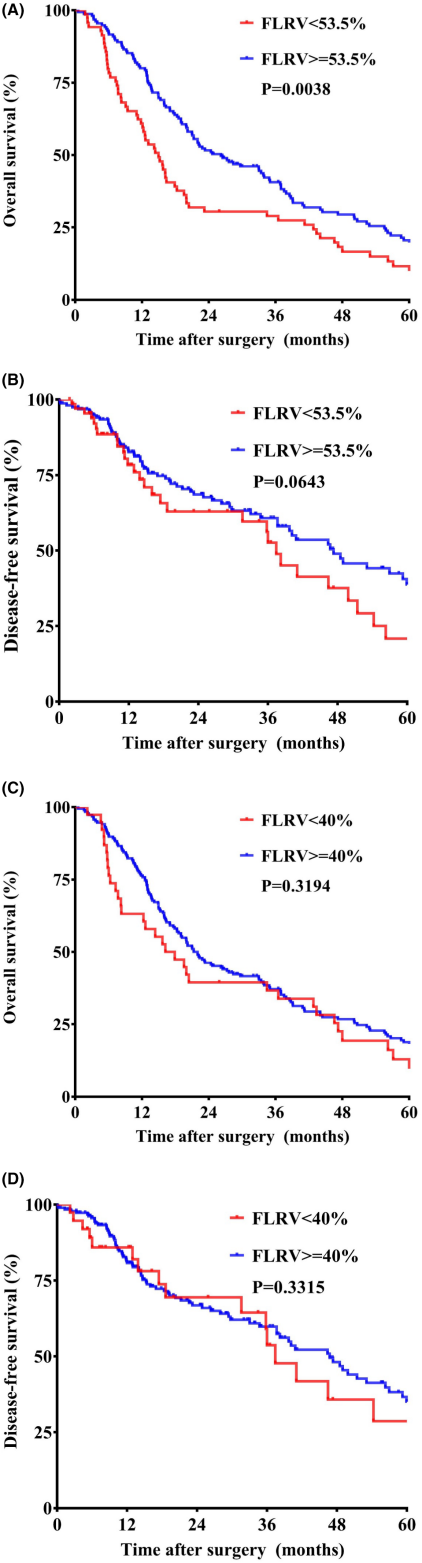
KM curves reflecting the survival difference based on different cut off values of FLRV. (A) OS based on FLRV of 53.5%; (B) DFS based on FLRV of 53.5%; (C) OS based on FLRV of 40%; (D) DFS based on FLRV of 40%.

### 3.5 Univariate and multi‐variate logistic regression for predictors on PHLF among resected HCCA patients with jaundice

Final results indicated that major hepatectomy (performed vs. not performed) (*p* < 0.0001), major vascular reconstruction (performed vs. not performed) (*p* = 0.0410), FLRV (≥53.5% vs. <53.5%) (*p* < 0.0001), FLRV (≥40% vs. <40%) (*p* = 0.003), PVE (Performed vs. not performed) (*p* = 0.0020), and TNM stage (I/II vs. III/IV) (*p* = 0.0300) were predictors of PHLF in resected HCCA patients with obstructive jaundice. Multi‐variate analysis indicated that FLRV (>53.5% vs. ≤53.5%) (*p* < 0.0001) was an independent predictor of PHLF. Moreover, major hepatectomy (performed vs. not performed) (*p* = 0.0610) was also a potential independent predictor of PHLF with a borderline *p* value 0.061 (Table 3).

**TABLE 3:** Univariate and multivariate logistic regression of predictors for postoperative liver failure.

Variables	Univariate *p*‐value	Multivariate analysis		
		OR	95%CI	Multivariate *p*‐value
Age (≥70 vs. <70)	0.2230			
Concurrent hepato‐atrophy (yes vs. no)	0.4190			
Major hepatectomy (performed vs. not performed)	<0.0001	0.156	0.022–1.093	0.0610
Major vascular reconstruction (performed vs. not performed)	0.0410	1.124	0.453–2.792	0.8010
FLRV (≥40% vs. <40%)	0.0030	0.697	0.107–4.535	0.7060
FLRV (≥53.5% vs. <53.5%)	<0.0001	83.124	18.601–371.456	<0.0001
Preoperative PVE (performed vs. not performed)	0.0020	1.636	0.205–13.091	0.6420
Preoperative biliary drainage (performed vs. not performed)	0.2200			
Preoperative cholangitis (yes vs. no)	0.5240			
Preoperative liver function (child A vs. child B)	0.5430			
TNM stage (I/II vs. III/IV)	0.0300	2.065	0.587–7.267	0.2590

Abbreviations: CI: confidence interval; FLRV: functional liver remnant volume; OR: odds ratio; PVE: portal vein embolization.

### 3.6 Univariate and multi‐variate cox regression of prognostic factors for OS of the entire cohort

As is recorded in Table 4, the results of univariate analyses indicated that Bismuth Type (I/II vs. III/IV) (*p* < 0.0001), FLRV (≥53.5% vs. <53.5%) (*p* = 0.0040), Surgical margin (positive vs. negative) (*p* = 0.002), Surgical margin width (<5 mm vs. ≥5 mm) (*p* = 0.050), T stage (T1–2 vs. T3–4, 8th AJCC) (*p* < 0.0001), Lymph node status (N− vs. N+, 8th AJCC) (*p* < 0.0001), perineural invasion (yes vs. no) (*p* = 0.0240) were all prognostic factors for OS of the entire cohort. Multi‐variate analysis indicated that Surgical margin (positive vs. negative) (*p* = 0.046), FLRV (≥53.5% vs. <53.5%) (*p* = 0.0470), T stage (T1–2 vs. T3–4, 8th AJCC) (*p* < 0.0001), and lymph node status (N− vs. N+, 8th AJCC) (*p* = 0.0020), and perineural invasion (yes vs. no) (*p* = 0.0330) were independent prognostic factors for OS of the entire cohort.

**TABLE 4:** Univariate and multivariate cox regression of prognostic factors for OS of the entire cohort.

Variables	Univariate *p* value	Multivariate analysis		
		HR	95%CI	Multivariate *p* value
Age (≥70 vs. <70)	0.6820			
Sex (male vs. female)	0.8010			
CA199 (<150 U/mL vs. ≥150 U/mL)	0.3080			
Bismuth type (I/II vs. III/IV)	<0.0001	1.286	0.898–1.843	0.1700
FLRV (≥53.5% vs. <53.5%)	0.0040	1.379	1.005–1.892	0.0470
Surgical margin (positive vs. negative)	0.0020	1.416	1.006–1.991	0.0460
Surgical margin width (<5 mm vs. ≥5 mm)	0.0050	0.782	0.560–1.093	0.1500
T stage (T1–2 vs. T3–4, 8th AJCC)	<0.0001	2.381	1.640–3.458	<0.0001
Lymph node status (N− vs. N+, 8th AJCC)	<0.0001	1.613	1.197–2.174	0.0020
Perineural invasion (yes vs. no)	0.0240	1.397	1.028–1.897	0.0330
Lymph‐vascular invasion (yes vs. no)	0.2050			

Abbreviations: AJCC, American Joint Committee on Cancer; CI, confidence interval; FLRV, functional liver remnant volume; HR, hazard ratio.

## 4 DISCUSSION

Our previous meta‐analysis has indicated that preoperative jaundice in in patients with gallbladder carcinoma was correlated with a significantly lower resectability rate and a higher incidence of perioperative mortalities.^18^ Similarly, in the surgical management of patients with HCCA, the majority of cases were presented with obstructive jaundice upon admission or surgery. Moreover, according to the latest NCCN guidelines, in order to acquire a clear margin, once the tumor invaded beyond the confluence of the right and left hepatic bile duct and involved the liver, the corresponding hemi‐hepatectomy would be unavoidable. In such a circumstance, owing to the hepatotoxicity resulted from high bilirubin level, HCCA patients who were about to receive curative surgery often took a high risk of PHLF and other severe postoperative morbidities, as well as perioperative mortalities. Therefore, it's quite necessary for clinicians to find optimal predictors in predicting PHLF.

Either for patients with hepatic malignancies or biliary malignancies, combined partial hepatectomy has always been performed in a high frequency and the potential predictors of PHLF has been widely discussed. Numerous studies have indicated that preoperative selective PVE could lead to a significantly decreased incidence of PHLF as well as early mortalities among surgically‐treated HCCA patients.^6^, ^9^ Others have revealed the predictive value of concurrent cholangitis in post‐hepatectomy liver insufficiency.^7^ Preoperative ICG R15 has been reported to be closely‐associated with PHLF and any other moderate‐to‐severe morbidities.^19^ External biliary drainage has been demonstrated to increase the risk of PHLF.^10^ Additionally, intraoperative major vascular resection and reconstruction has also been demonstrated to be closely‐associated with liver disfunction.^11^ The incidence of major vascular reconstruction of our cohort in jaundiced patients was 29% and patients with obstructive jaundice shared a significantly higher incidence of major vascular reconstruction than those without it (*p* = 0.0320). Intraoperative major vascular (portal vein, hepatic artery, or major hepatic veins) resections and reconstructions are generally performed in patients with an obvious outflow obstruction. The venous wall was often thin and fragile and it is often difficult to reconstruct and re‐anastomosis the broken ends of venous vessels, which would lead to an uncontrolled intraoperative bleeding as well as warm ischemia time, causing severe ischemia‐resulted injury to our liver.^20^, ^21^, ^22^, ^23^ The massive intraoperative bleeding would furtherly increase the difficulty of surgery, which would significantly prolong operative time and increase intraoperative blood loss. Besides, intraoperative long‐time hepatic inflow occlusion is often required in the process of major vascular reconstruction, which would furtherly aggravate ischemia‐mediated liver injury furtherly. Moreover, many other postoperative risk factors, such as severe abdominal infectious disease, would also contribute a lot to the development of secondary PHLF. However, this issue has been rarely explored.

Although various invasive or non‐invasive predictors have been reported and discussed, FLRV has always been the most applicable modality in evaluating PHLF for patients who were about to receive a combined radical resection. Lee JW et al indicated a high predictive accuracy in PHLF when it combined with volume‐to‐body weight.^8^ FLRV has also been reported as independent predictor of PHLD in patients with cirrhotic HCC.^24^ The defects of FLRV have also been discussed and previous studies have showed its inferiority versus Future liver remnant function (FLRF).^25^, ^26^ However, we cannot deny the fact that FLRV still served as the corner stone in predicting PHLF that FLRF was calculated from FRL: FLRF = FLRV% × total liver function. To date, few researchers have paid attention on the more specific criteria of FLRV in different situations, especially in jaundiced patients with HCCA. Considering the more complex and severe burden to liver among jaundiced HCCA patients as mentioned above, the classical and conventional criteria of FLRV <40% might be not applicable to some extent. Consequently, based on our single‐center experience, we focused on jaundiced HCCA patients and have put forward a new standard of FLRV. The newly‐detected FLRV level was correlated with a much higher predictive accuracy in PHLF versus the old standard (FLRV: 40%) and has been demonstrated as an independent predictor of PHLF. Further analyses regarding its impact on the clinic‐pathological features and long‐term survival has been performed. The final results indicated that the newly‐acquired FLRV (53.5%) could better help clinicians to stratify patients with different postoperative outcomes, especially the occurrence of postoperative major morbidities versus conventional FLRV (40%) (*p* < 0.0001 vs. *p* = 0.133, Table 2). Moreover, in the subsequent survival analysis, patients with an estimated FLRV lower than 53.5% even shared a worse OS than those with FLRV higher than 53.5% while the phenomenon was not obvious for FLRV of 40%. These promising findings might help clinicians to choose more favorable therapeutic regimes for HCCA patients who were about to receive curative surgery with more satisfying postoperative and survival outcomes. More deeply, we furtherly evaluated the predictive accuracy of FLRV in jaundiced HCCA patients based on the perform of major hepatectomy. As is illustrated in Figure S1, among patients who received major hepatectomy, FLRV showed an extremely lower predictive accuracy versus those who did not receive major hepatectomy (AUC 0.52 vs. AUC 0.86). One of the reasons might be that major hepatectomy also served as an independent predictor of PHLF with a borderline *p* value (*p* = 0.061) and the effect of FLRV was confounded.

Current study should be interpreted with several limitations. Firstly, our study is a single‐center study and only reflected general condition of Asian populations. Secondly, our cohort came across nearly two decades and the development in perioperative management would also impact a lot on the occurrence of PHLF. Thirdly, if various geographical and genetic characteristics could be incorporated, the results would be more validating. However, the inadequate original data hindered us from further exploration. In such a case, a multi‐center studies with geographical, racial and genetic data included, accompanied with training group and validation group, would be furtherly required. Fourth, the correlation between tumor diameter and FLRV or PHLF should also be furtherly evaluated. The larger the tumor volume, the higher the likelihood of tumor invasion into the liver or the tumor extending beyond the hilar bile duct confluence, resulting in a greater extent of liver resection needed to achieve R0 resection. However, in our tumor database, only a small portion of patients have clear tumor diameter information. Therefore, the above analysis is not feasible in our single‐center study. This issue requires further exploration in future larger sample size, multicenter studies. Finally, many other potential influencing factors, such as preoperative comorbidities, still need to be analyzed furtherly. However, the inadequate original date hindered us from further exploration.

## 5 CONCLUSION

FLRV (continuous data) had a high predictive accuracy in jaundiced patients in predicting PHLF and the newly‐acquired cut off value (FLRV: 53.5%) showed an extremely high predictive accuracy (AUC: 0.81) than conventional FLRV <40% (AUC: 0.60) (*p* < 0.05). Moreover, its impact on various postoperative outcomes, especially the occurrence of major morbidities, as well as long‐term survival has also been revealed, which might help clinicians to stratify patients with different therapeutic modalities and outcomes. Future multi‐center studies with more parameters included, such as geographical, racial and genetic data, for training and validation are required for further validation.

## AUTHOR CONTRIBUTIONS

**Tian‐Run Lv:** Conceptualization (lead); data curation (lead); formal analysis (lead); resources (lead); software (lead); supervision (lead); validation (lead); visualization (lead); writing – original draft (lead); writing – review and editing (lead). **Wen‐Jie Ma:** Conceptualization (equal); data curation (equal); formal analysis (equal); writing – original draft (equal); writing – review and editing (equal). **Fei Liu:** Conceptualization (equal); data curation (equal); formal analysis (equal); visualization (equal); writing – review and editing (equal). **Hai‐Jie Hu:** Funding acquisition (lead). **Yan‐Wen Jin:** Resources (equal); software (equal); supervision (equal); validation (equal); visualization (equal); writing – review and editing (equal). **Fu‐Yu Li:** Methodology (equal); project administration (lead); writing – review and editing (equal).

## FUNDING INFORMATION

This study was supported by 1.3.5 project for disciplines of excellence, West China Hospital, Sichuan University (ZYJC21046); 1.3.5 project for disciplines of excellence‐Clinical Research Incubation Project, West China Hospital, Sichuan University (2021HXFH001); China Telecom Sichuan Company Biliary Tract Tumor Big Data Platform and Application Phase I R&D Project (312230752). National Natural Science Foundation of China for Young Scientists Fund (82203650, 82203782); Sichuan Natural Science Foundation (2024NSFSC0742, 2024NSFSC1949); Sichuan University‐Sui Ning School‐local Cooperation project (2022CDSN‐18).

## CONFLICT OF INTEREST STATEMENT

All authors declare have no conflicts of interest to disclose.

## Supporting information


Figure S1.

Figure S2.

Table S1.

## Data Availability

All original data were extracted from cancer database of our hospital and can be provided if required.
