# Supplementary material for: Is conventional functional liver remnant volume higher than 40% still sufficient to prevent post‐hepatectomy liver failure in jaundiced patients with hilar cholangiocarcinoma? A single‐center experience in China
Source: Cancer Med. 2024 Jul 5;13(13):e7342. doi: 10.1002/cam4.7342 (PMC11224912; doi:10.1002/cam4.7342)
Supplement: Supplementary file 1 — Figure S1. [file CAM4-13-e7342-s002.docx]

**Figure S1**

**Figure S1A**


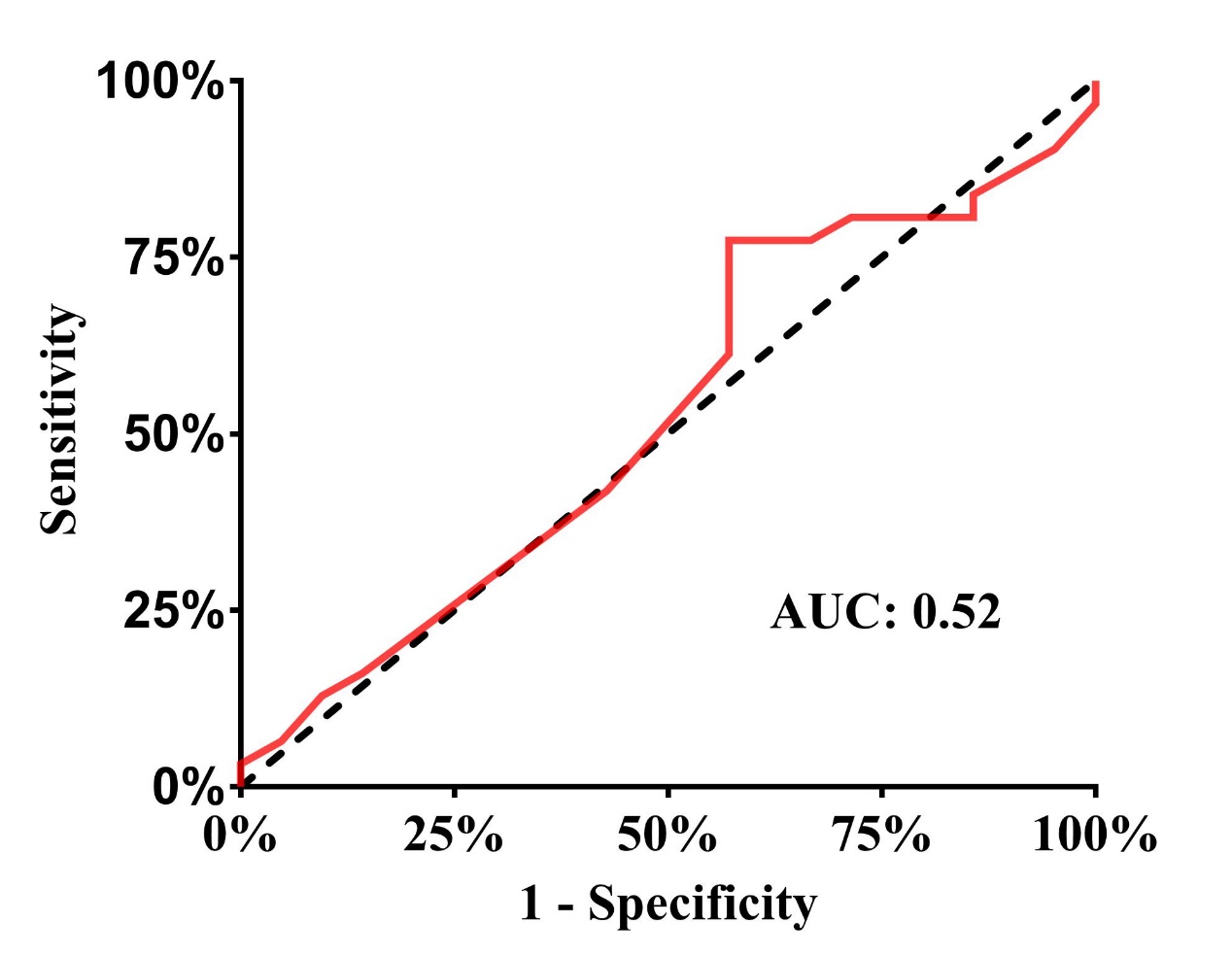


**Figure S1B**


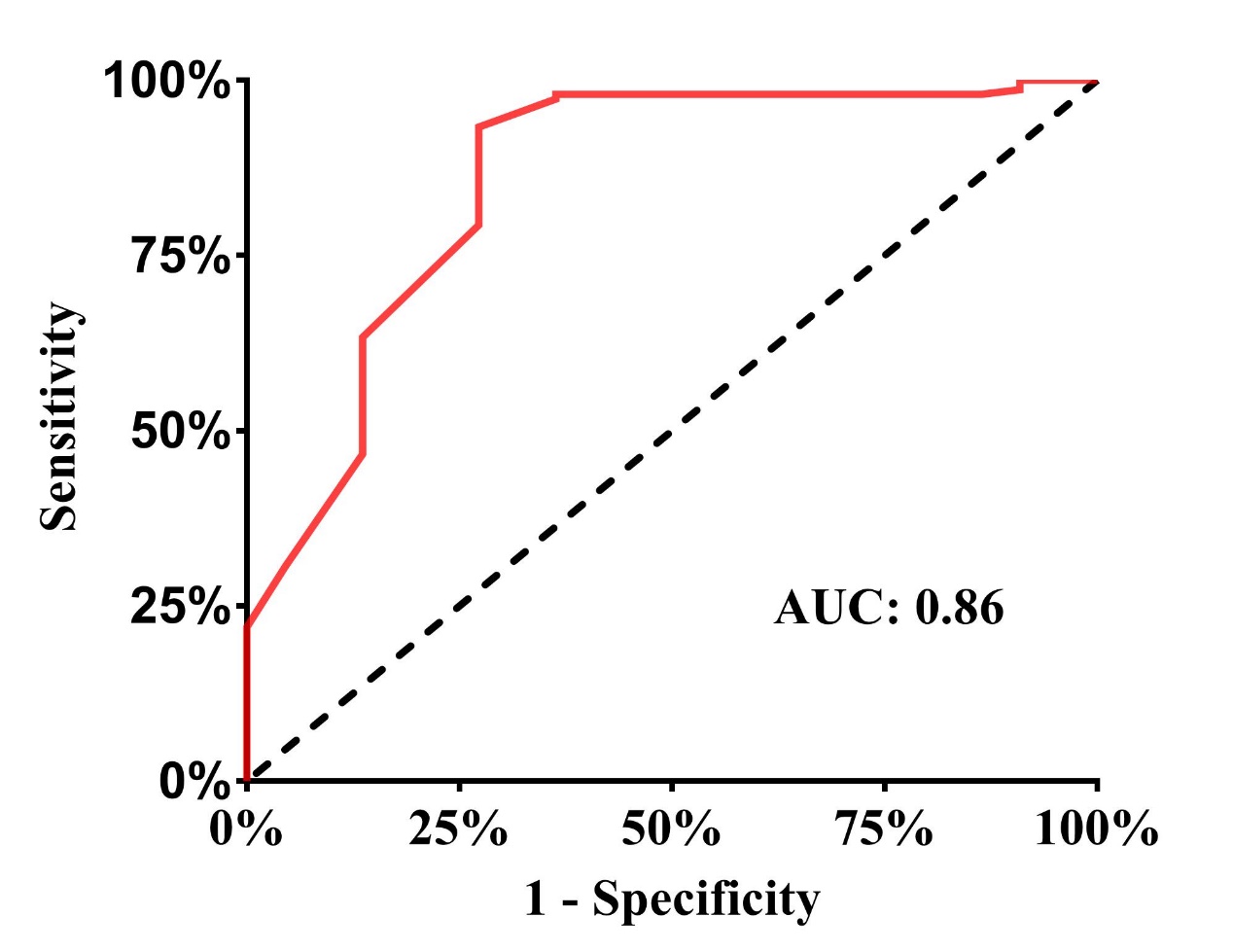


**Figure S1** ROC curves reflecting the predictive accuracy of FLRV in jaundiced HCCA patients based on the perform of major hepatectomy. A, FLRV in patients who received major hepatectomy; B, FLRV in patients who did not receive major hepatectomy.
