# Supplementary material for: Is conventional functional liver remnant volume higher than 40% still sufficient to prevent post‐hepatectomy liver failure in jaundiced patients with hilar cholangiocarcinoma? A single‐center experience in China
Source: Cancer Med. 2024 Jul 5;13(13):e7342. doi: 10.1002/cam4.7342 (PMC11224912; doi:10.1002/cam4.7342)
Supplement: Supplementary file 2 — Figure S2. [file CAM4-13-e7342-s003.docx]

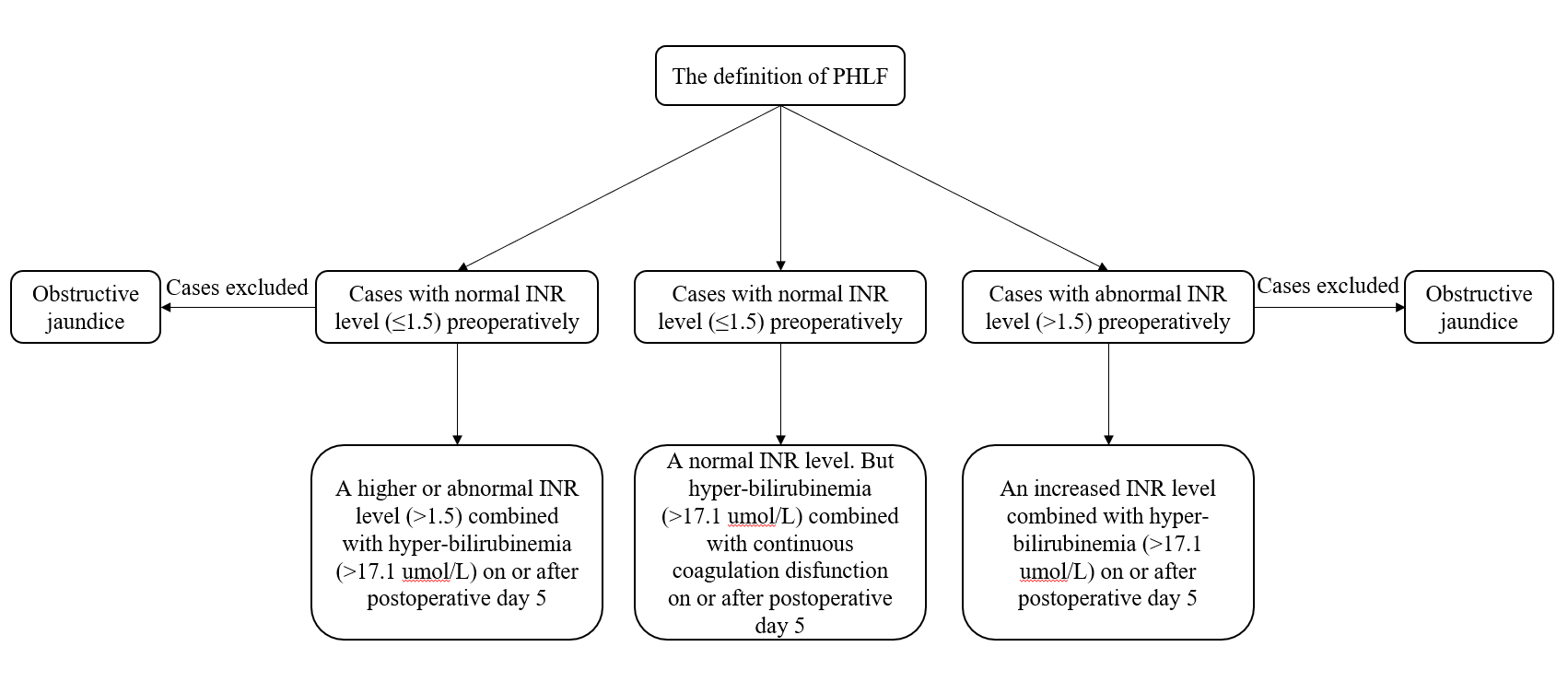


**Figure S2** Flowchart illustrating the definition of PHLF of current study. PHLF: post-hepatectomy liver failure.
