# Supplementary material for: Is conventional functional liver remnant volume higher than 40% still sufficient to prevent post‐hepatectomy liver failure in jaundiced patients with hilar cholangiocarcinoma? A single‐center experience in China
Source: Cancer Med. 2024 Jul 5;13(13):e7342. doi: 10.1002/cam4.7342 (PMC11224912; doi:10.1002/cam4.7342)
Supplement: Supplementary file 3 — Table S1. [file CAM4-13-e7342-s001.docx]

| **Table S1 Logistic regression evaluating the significance of FLRV (continuous data) in PHLF** | | | | |  |
| --- | --- | --- | --- | --- | --- |
| **Variables** | **Univariate P value** | **Multivariate analysis** | | |  |
|  |  | **OR** | **95%CI** | **Multivariate P value** |  |
| **Age (>=70 vs ＜70)** | 0.2230 |  |  |  |  |
| **Concurrent Hepato-atrophy (Yes vs no)** | 0.4190 |  |  |  |  |
| **Major Hepatectomy (Performed vs not performed)** | <0.0001 | 0.032 | 0.005-0.211 | <0.0001 |  |
| **Major Vascular Reconstruction (Performed vs not performed)** | 0.0410 | 1.281 | 0.544-3.015 | 0.5710 |  |
| **FLRV (Continuous data)** | <0.0001 | 0.817 | 0.758-0.881 | <0.0001 |  |
| **Preoperative PVE (Performed vs not performed)** | 0.0020 | 11.491 | 2.845-46.418 | 0.0010 |  |
| **Preoperative biliary drainage (Performed vs not performed)** | 0.2200 |  |  |  |  |
| **Preoperative cholangitis (Yes vs no)** | 0.5240 |  |  |  |  |
| **Preoperative liver function (Child A vs Child B)** | 0.5430 |  |  |  |  |
| **TNM stage (I/II vs III/IV)** | 0.0300 | 2.477 | 0.718-8.540 | 0.1510 |  |
| OR: odds ratio; CI: confidence interval; FLRV: functional liver remnant volume; PVE: portal vein embolization; PHLF: post-hepatectomy liver failure | | | | |  |
|  |  |  |  |  |  |
